# Biological sex impacts COVID-19 outcomes

**DOI:** 10.1371/journal.ppat.1008570

**Published:** 2020-06-22

**Authors:** Sabra L. Klein, Santosh Dhakal, Rebecca L. Ursin, Sharvari Deshpande, Kathryn Sandberg, Franck Mauvais-Jarvis

**Affiliations:** 1 W. Harry Feinstone Department of Molecular Microbiology and Immunology, The Johns Hopkins Bloomberg School of Public Health, Baltimore, Maryland, United States of America; 2 Department of Biochemistry and Molecular Biology, The Johns Hopkins Bloomberg School of Public Health, Baltimore, Maryland, United States of America; 3 Departments of Medicine and Nephrology & Hypertension, Georgetown University, Washington, DC, United States of America; 4 Diabetes Discovery & Sex-Based Medicine Laboratory, Section of Endocrinology, John W. Deming Department of Medicine, Tulane University School of Medicine, New Orleans, Louisiana, United States of America; 5 Southeast Louisiana Veterans Health Care System Medical Center, New Orleans, Louisiana, United States of America; University of Pittsburgh, UNITED STATES

## Abstract

The current novel coronavirus disease 2019 (COVID-19) pandemic is revealing profound differences between men and women in disease outcomes worldwide. In the United States, there has been inconsistent reporting and analyses of male–female differences in COVID-19 cases, hospitalizations, and deaths. We seek to raise awareness about the male-biased severe outcomes from COVID-19, highlighting the mechanistic differences including in the expression and activity of angiotensin-converting enzyme 2 (ACE2) as well as in antiviral immunity. We also highlight how sex differences in comorbidities, which can be associated with both age and race, impact male-biased outcomes from COVID-19.

We are in the midst of a pandemic. Many of us predicted that the next “100 year pandemic” would be caused by an influenza A virus, like the H1N1 virus that caused the 1918 influenza pandemic. Instead, the current pandemic is caused by a novel β-coronavirus, the severe acute respiratory syndrome coronavirus 2 (SARS-CoV2). Currently, there are almost 2 million cases and over 100,000 deaths worldwide from the disease caused by this virus, called the novel coronavirus disease 2019 (COVID-19). Like the 1918 influenza pandemic [1], men are at greater risk of more severe COVID-19 outcomes than women, with both sex (i.e., biological differences) and gender (i.e., sociocultural and behavioral differences) playing fundamental roles.

The initial reports from China, followed by data from several countries in Europe, have highlighted that there are roughly similar numbers of confirmed SARS-CoV2 cases between men and women. The severity of COVID-19, as measured by hospitalization, admission to intensive care units, and rates of fatality, however, has consistently been 2-fold greater for men than women [2], with the Global Health 50/50 research initiative providing real-time sex-disaggregated data from most countries worldwide [3]. Unfortunately, despite the United States currently having the most COVID-19 cases in the world, considerably less attention has been paid to sex-disaggregation of data than in Europe and China.

We took this opportunity to evaluate the current situation in the US to both determine if similar patterns of male–female differences are observed and to document which states are or are not disaggregating and analyzing data by sex. As of this writing, 26 states have more than 2,000 confirmed cases (https://www.worldometers.info) and from these states, only three (Louisiana, New Jersey, and Pennsylvania) have not sex-disaggregated cases of COVID-19. New York has the greatest number of COVID-19 cases in the US and is an epicenter of this pandemic. Of the data from the remaining 23 states, 7 states replicate the epidemiological pattern seen in New York City (NYC) (**Fig 1**) and elsewhere in the world [3], in which numbers of COVID-19 cases are similar between men and women. The other 16 states, however, suggest a female-bias exists in COVID-19 cases (i.e., 1 to 0.9/0.8 male to female ratio). This includes Washington state, which is another epicenter of the COVID-19 pandemic in the US. Of 167 COVID-19 cases from a Washington state long-term care facility, a majority of cases were women (68% of residents and 76% of healthcare workers) [4]. The total number of men and women among facility residents and healthcare workers was not provided and with women living longer than men and being more likely to work as healthcare providers [5], gender-associated factors may be involved [5].

**Fig 1:**
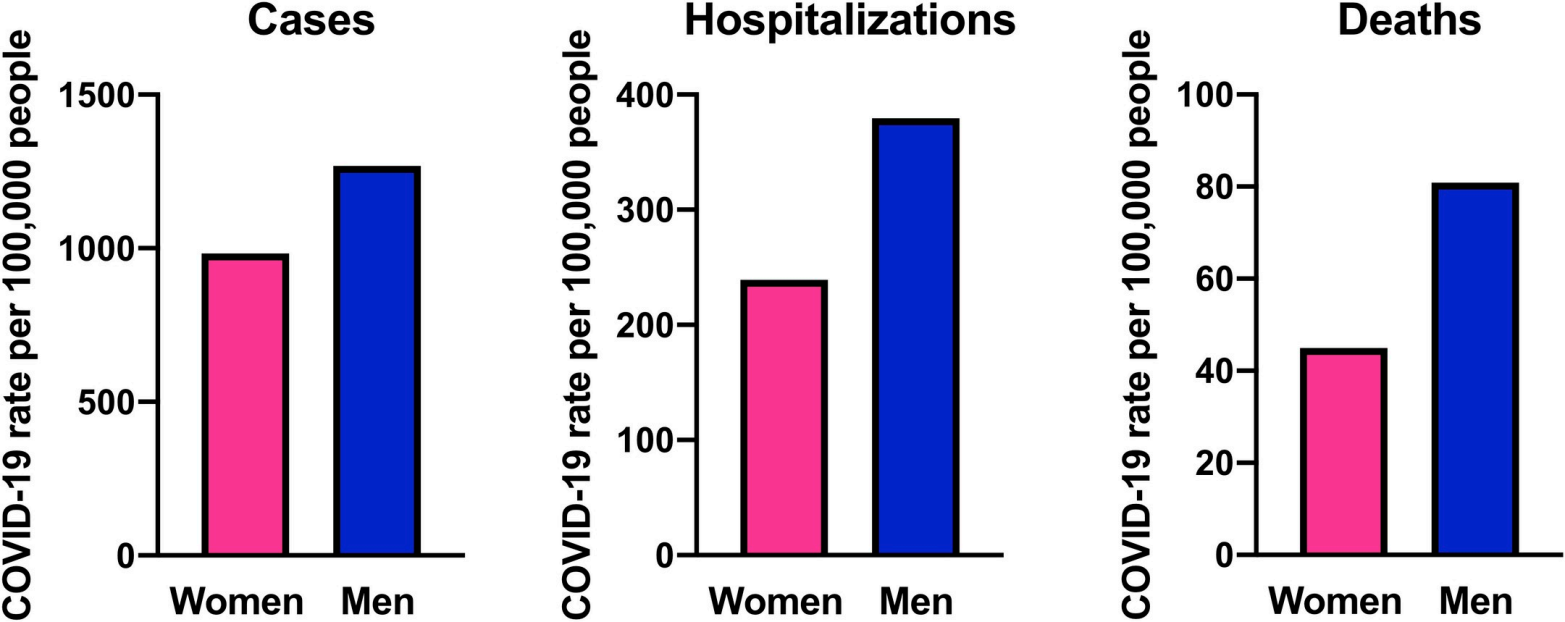
Sex-disaggregated numbers of COVID-19 cases, hospitalizations, and deaths per 100,000 people in NYC. Data were accessed from https://www1.nyc.gov/site/doh/covid/covid-19-data.page on April 11, 2020. COVID-19, novel coronavirus disease 2019; NYC, New York City.

Of the 26 states analyzed, only two counties within two different states (i.e., NYC and Bucks County, Pennsylvania) have reported rates of hospitalization broken down by sex, and both report greater rates of hospitalization from COVID-19 among men than women (**Fig 1**). Lastly, of the 26 states with more than 2,000 confirmed COVID-19 cases, only 13 (New York, Michigan, California, Illinois, Texas, Washington, Connecticut, Indiana, Colorado, Ohio, North Carolina, Wisconsin, and Alabama) have disaggregated fatalities from COVID-19 by sex and consistently show that fatality rates are 2-fold greater for men than women (**Fig 1**). Gender-associated factors have been reviewed elsewhere [2, 5]; thus, we seek to focus on biological mechanisms that could impact male–female differences in severe COVID-19 outcomes to call attention to sex-associated factors that could potentially provide novel insights into therapeutic interventions.

Angiotensin-converting enzyme 2 (ACE2) is a monocarboxpeptidase that counteracts the vasoconstrictor effects of angiotensin (Ang)-(1–8) by converting this octapeptide hormone to the vasodilator heptapeptide Ang-(1–7) [6]. In 2003, ACE2 was found to be the SARS-CoV (i.e., the virus that caused the 2002 to 2003 SARS outbreak) receptor [7, 8], with SARS-CoV2 binding ACE2 with even higher affinity [9]. ACE2 is expressed primarily in the kidney, heart, and testes, with the highest expression occurring in the kidney; it is, however, also expressed in the lungs at much lower levels [10]. The SARS-CoV2 virus S1 spike protein binds to the ACE2 receptor in alveolar epithelial cells of the lungs. ACE2 protein is expressed in a sex-specific manner in the mouse kidney; male mice have nearly 2-fold higher levels of renal ACE2 protein than female mice [11]. Furthermore, using the four core genotype mouse model in which gonadal sex (ovaries versus testes) is separated from the sex chromosome complement (XX versus XY) [12], we found renal ACE2 activity was greater in the male kidney regardless of the sex chromosome complement [11]. This sex difference in renal ACE2 activity was driven by estradiol reducing ACE2 activity regardless of the sex chromosome complement. These findings have implications for the observed sex differences in COVID-19 outcomes. It will be important to study the sex-specific regulation of ACE2 in the lung and other tissues involved in COVID-19 pathogenesis including the heart and brain [6] and also to investigate whether estrogens protects women from COVID-19 by reducing the expression levels of the receptor for the SARS-CoV2 virus.

The innate recognition and response to viruses as well as downstream adaptive immune responses during viral infections also differ between females and males [13]. We and others have illustrated that females generally mount greater inflammatory, antiviral, and humoral immune responses than males during viral infections [14], which contributes to better clearance of viruses, including SARS-CoV [15]. Enhanced immunity in females can, however, also result in greater immunopathology and tissue damage at later stages of viral disease, such as during influenza A virus infection [16]. To date, we have only identified two COVID-19 studies that have disaggregated and analyzed immunological outcome data by sex. In a published study of 168 patients with severe COVID-19 in Wuhan, China, it was reported that men were significantly more likely to remain hospitalized and die and less likely to be discharged from the hospital during the study period than women [17]. The male–female difference was most pronounced among individuals 60 years of age and older. In this study, the neutrophil to lymphocyte ratio and serum C-reactive protein concentrations were twice as high in male as in female COVID-19 patients, as well as in patients who died compared with patients who were discharged from the hospital (not disaggregated by sex) [17]. These data suggest that inflammatory immune responses and cell counts might be more elevated in men and associated with worse outcomes from COVID-19 than in women.

Mounting evidence suggests that humoral immune responses can be measured not only to confirm exposure to SARS-CoV2 but also to assess adaptive immune responses necessary for clearance of SARS-CoV2. As a result, convalescent plasma transfer studies are underway for compassionate care of severe COVID-19 patients [18], without, however, consideration of the sex of the donor. In a not yet peer-reviewed study of 331 patients with confirmed SARS-CoV2 infections in Wuhan, China, anti–SARS-CoV2 immunoglobulin G (IgG) responses were measured and compared among patients with either clinically diagnosed mild or severe disease. The sex distribution of recovering cases was 36% and 65% for men and women, respectively [19]. Among patients with mild COVID-19, anti–SARS-CoV2 IgG titers were similar between the sexes. In contrast, among patients with severe disease, women exhibited greater antibody responses than men, with production of antibodies at earlier phases of disease suggesting one possible immunological mechanism mediating better recovery from COVID-19 in women than men [19].

Development of mouse models for SARS-CoV2 will be instrumental for mechanistically assessing the causes of sex differences in the pathogenesis of COVID-19. In a mouse model of SARS-CoV infection, female mice had lower virus titers and less severe pulmonary damage from monocyte–macrophage infiltration and cytokine production, resulting in lower mortality in female (20%) compared with male (80%) mice [15]; a sex distribution similar to that observed in human SARS [20]. Notably, the endogenous production of estradiol in female mice was important for this protection.

Comorbidities that are associated with more severe outcomes from COVID-19 in the US, include diabetes, obesity, hypertension, heart disease, chronic kidney disease, and chronic pulmonary disease. Notably, diabetes, obesity, and hypertension are the top three conditions associated with fatal COVID-19 cases in China and Italy [4, 21–23]. As of April 14, 2020, in New York, hypertension accounted for 56.8% and diabetes 42.4% of fatal cases [24]. In Louisiana, where New Orleans is the epicenter by death rate per capita, hypertension accounted for 59.8%, diabetes 38.1% and obesity 22.3% of fatal cases [25]. To date, no study has reported whether these comorbidities are influenced by sex or gender in COVID-19 patients. Biological (sex) as well as behavioral (gender) factors contribute to differences between men and women in these comorbidities [26]; the sex and gender-associated factors that underlie these comorbidities, however, have not been evaluated in context of COVID-19. There also has been no consideration about how sex intersects with age and race to further increase risk of severe COVID-19 outcomes in men, despite observations illustrating that older aged individuals [27] and African Americans [28] are also at risk for severe COVID-19 outcomes. For these reasons, we call on clinicians and epidemiologists to report data pertaining to comorbidities associated with COVID-19 disaggregated by sex, age, and race. We also emphasize the importance of considering the biological variable of sex when conducting basic science studies of COVID-19.
